# Drug triggered pruritus, rash, papules, and blisters – is AGEP a clash of an altered sphingolipid-metabolism and lysosomotropism of drugs accumulating in the skin?

**DOI:** 10.1186/s12944-021-01552-3

**Published:** 2021-11-08

**Authors:** Markus Blaess, Lars Kaiser, Oliver Sommerfeld, René Csuk, Hans-Peter Deigner

**Affiliations:** 1 Institute of Precision Medicine, Medical and Life Sciences Faculty, Furtwangen University, Jakob-Kienzle-Str. 17, D-78054 Villingen-Schwenningen, Germany; 2 Institute of Pharmaceutical Sciences, University of Freiburg, Albertstraße 25, D-79104 Freiburg, Germany; 3 Department of Anaesthesiology and Intensive Care Medicine, Jena University Hospital, Am Klinikum 1, D-07747 Jena, Germany; 4 Organic Chemistry, Martin-Luther-University Halle-Wittenberg, Kurt-Mothes-Straße 2, D-06120 Halle (Saale), Germany; 5 EXIM Department, Fraunhofer Institute IZI, Schillingallee 68, D-18057 Leipzig, Rostock, Germany; 6 Faculty of Science, Associated member of Tuebingen University, Auf der Morgenstelle 8, D- 72076 Tübingen, Germany

**Keywords:** Lysosomotropism, Sphingolipid metabolism, Elongation of very long-chain fatty acids, Pruritic papules, Pruritus, Lysosome, Metabolites, Approved drugs, Adverse reactions of the skin and subcutaneous tissue, Photosensitivity

## Abstract

Rash, photosensitivity, erythema multiforme, and the acute generalized exanthematous pustulosis (AGEP) are relatively uncommon adverse reactions of drugs. To date, the etiology is not well understood and individual susceptibility still remains unknown. Amiodarone, chlorpromazine, amitriptyline, and trimipramine are classified lysosomotropic as well as photosensitizing, however, they fail to trigger rash and pruritic papules in all individuals. Lysosomotropism is a common charcteristic of various drugs, but independent of individuals. There is evidence that the individual ability to respond to external oxidative stress is crosslinked with the elongation of long-chain fatty acids to very long-chain fatty acids by ELOVLs. ELOVL6 and ELOVL7 are sensitive to ROS induced depletion of cellular NADPH and insufficient regeneration via the pentose phosphate pathway and mitochondrial fatty acid oxidation. Deficiency of NADPH in presence of lysosomotropic drugs promotes the synthesis of C_16_-ceramide in lysosomes and may contribute to emerging pruritic papules of AGEP. However, independently from a lysosomomotropic drug, severe depletion of ATP and NAD(P)H, e.g., by UV radiation or a potent photosensitizer can trigger likewise the collapse of the lysosomal transmembrane proton gradient resulting in lysosomal C_16_-ceramide synthesis and pruritic papules. This kind of papules are equally present in polymorphous light eruption (PMLE/PLE) and acne aestivalis (Mallorca acne). The suggested model of a compartmentalized ceramide metabolism provides a more sophisticated explanation of cutaneous drug adverse effects and the individual sensitivity to UV radiation. Parameters such as pKa and ClogP of the triggering drug, cutaneous fatty acid profile, and ceramide profile enables new concepts in risk assessment and scoring of AGEP as well as prophylaxis outcome.

Rash, urticaria, photosensitivity, erythema multiforme, and the more severe cutaneous adverse reactions acute generalized exanthematous pustulosis (AGEP), drug reaction with eosinophilia and systemic symptoms (DRESS), and toxic epidermal necrolysis (TEN) are relatively uncommon adverse effects of applied drugs (Table 1) [1]. Non-scarring, pruritic, erythematous papules, papulovesicles, vesicles or plaques are, however, likewise characteristics of polymorphous light eruption (PMLE/PLE) that can be triggered with delayed onset by UV light rather than by a drug [2].

**Table 1:**
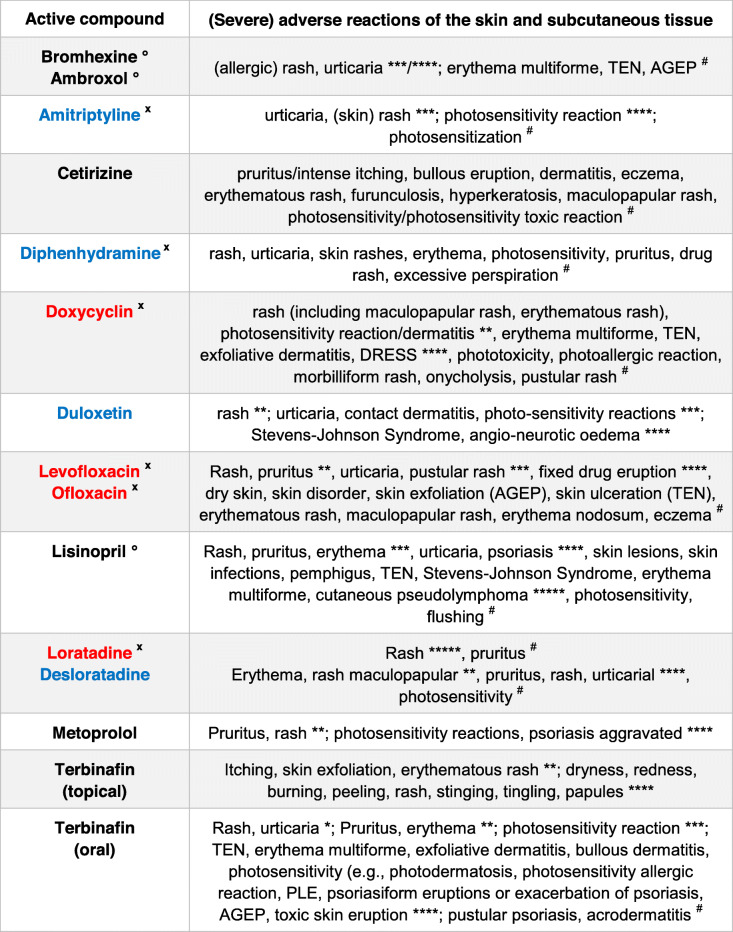
Compilation of severe cutaneous adverse reactions (SCAR) of the skin and subcutaneous tissue of various approved drugs and their metabolites with supposed lysosomotropism (°), confirmed lysosomotropism (blue), no lysosomotropism (red), and photosensitizing (^**x**^) [3–7] according to their human medicine european public assessment report (EPAR) product information [8] and https://www.drugs.com. Frequency (CIOMS classification) is given by very common (≥ 1/10) (*), frequent/common (> 1/100, < 1/10) (**), infrequent/uncommon (> 1/1.000, < 1/100) (***), rare (> 1/10.000, < 1/1.000) (****), very rare (< 1/10.000) (*****), and frequency not reported (^#^)

AGEP and PLE are both recurrent and share the common feature of rash with papules and blistering occurring with a delayed onset to the beginning of the drug application or the exposure to ultraviolet (UV) radiation, e.g., mostly the sun, and resolve completely without scarring if the trigger is stopped. Consequently, in the event of occurrence, the triggering drug should be discontinued (AGEP) or intensive sunbaths should be avoided and the use of sunscreens is mandatory (PLE). Topical corticosteroids are suggested in the acute stage, especially in inflammatory or pruritic areas of the skin. In severe cases oral corticosteroids may be considered [1, 2, 9].

PLE, photosensitivity related to a drug or cosmetics, and severe cutaneous adverse effects do not affect all individuals, susceptibility and severity vary significantly [2, 3, 10]. To date, the etiology is not well understood. Although there are various explanations existing to elucidate the photosensitization by drugs [3, 10] and PLE [2], however, the determination of the individual response and susceptibility still remains unknown. The route of delivery, the chemical characteristics of the drug, the individual, and the mechanisms causing the abnormal photosensitization contribute to the heterogeneity of presentation and clinical features [10]. Moreover, there is still no reliable method or biomarker existing to estimate the susceptibility to cutaneous adverse effects of drugs, to sunlight, and UV radiation.

## Antihistaminic therapy of hives and PLE can trigger AGEP

The hallmark of AGEP is an edematous diffuse erythema with the rapid appearance of multiple, sterile non-follicular pustules [1, 11]. Hives, however, involve pruritic, raised skin wheals, which may or may not be edematous [12]. Tissue swelling, vasodilatation, and the formation of wheals can be prevented by blocking the participating H_1_-receptors using antihistamines such as dimetidine and loratadine/desloratadine. According to their product information, H_1_-antihistamines can trigger AGEP with varying frequency [8], depending on individual susceptibility to cutaneous adverse drug reactions. AGEP related papules and blistering are independent of mast cells and possibly proceed via neutrophils. So far, the pathophysiology has remained largely unclear [13]. With discontinuation of the triggering drug, the affected areas recover without scarring. Although cutaneous and oral therapy with H_1_-antihistamines fail short of the relief of itching and rash, they were suggested as an option in treatment of PLE [14]. Using antihistamines in allergic reactions (e.g., insect bites) poses the risk of emerging pruritic papules (AGEP). Erythema and emerging papules induced by loratadine is attributed, in part, to its photosensitization [3]. Although cetirizine is not classified as photosensitizing, it is likewise capable of triggering pruritic papules.

These findings suggest that photosensitizing characteristics of a drug are obviously not the key trigger. Consequently, the question arises which preconditions trigger papules and rash in the event of concurrent in presence of cetirizine or loratadine. We try to provide an insight at the subcellular level by considering the interaction of the drugs with changes in sphingolipid metabolism.

## Cellular pathogenesis of AGEP

Currently, AGEP is classified as type IVd hypersensitivity reactions with a T-cell mediated neutrophilic inflammation after exposure to a drug or metabolite(s) [15]. After binding to host proteins and forming drug epitopes, the drug related epitopes are incorperated by antigen-presenting cells (APCs) to activate specific CD4 + and CD8 + cells (drug specific T-cells). The typical non-follicular sterile pustules of AGEP arise from activated, into dermis and epidermis migrated drug specific CD8 + cells. Activated, drug specific T-cells induce apoptosis of keratinocytes via the release of cytotoxic proteins (granzyme B, perforin, and Fas ligand), leading to tissue destruction and formation of sub-corneal vesicles [15–17]. In subcorneal pustules, epidermis, and dermis of AGEP IL-17A/F-expressing cells are significantly increased. Neutrophils and mast cells are considered to be the predominant cellular sources of IL-17A/F, found in subcorneal pustules and, to a lesser extent, in the epidermis near pustules and the upper dermis [18]. The release of IL-17 triggers epithelial cells such as keratinocytes to release the potent neutrophil chemoattractant CXCL8 (IL-8) and the granulocyte colony-stimulating factor (G-CSF), a survival factor for neutrophils [19]. Furthermore, CD4 + cells within these vesicles release CXCL8, granulocyte–macrophage colony-stimulating factor (GM-CSF) to prevent apoptosis of the recruited neutrophils, and interferon (IFN) gamma to promote further release of CXCL8 from surrounding keratinocytes [15, 20]. The pronounced chemotaxis of neutrophils into vesicles mediates the transformation of sub-corneal vesicles into sterile pustules [16, 17]. Thus, the histological and cellular level plus the role of the innate immune cells in the pathogenesis of AGEP is already well characterized. However, there is a lack of knowledge about the pathogenesis at molecular and subcellular level. The fact that long-chain ceramides can trigger exocytosis and endocytosis [21, 22] and are supposed to participate in incorporation and presentation of drug related epitopes by APCs to activate specific CD4 + and CD8 + cells (drug specific T-cells) vesicles together with lysosomes [15–17, 23, 24] suggests a participation with lysosomal sphingolipid metabolism.

## Lysosomotropism and AGEP

**Fig. 1:**
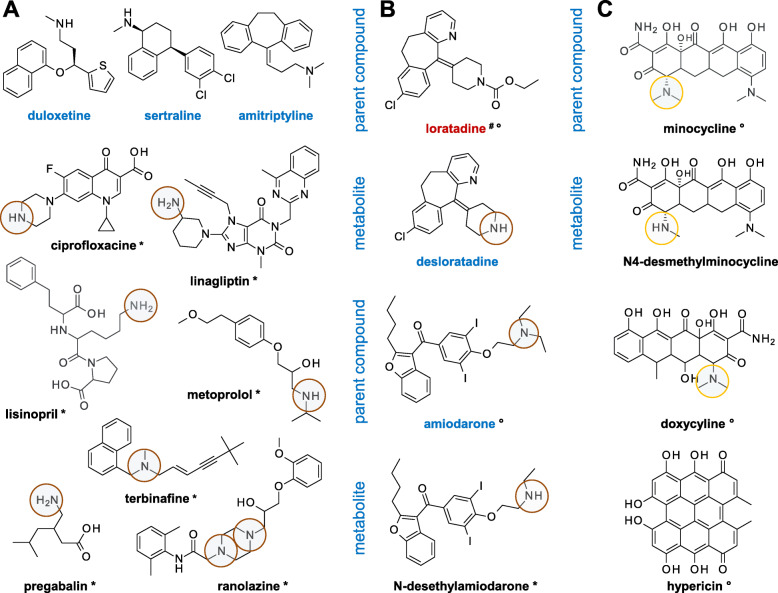
Lysosomotropism and photosensitizing. Various drugs with supposed lysosomotropism (*), confirmed lysosomotropism (blue), no lysosomotropism (red) [6, 7], and photosensitizing (°) [3] with characteristic cutaneous adverse reactions (rash, urticaria, pruritus, and AGEP) according to their human medicine european public assessment report (EPAR) product information [8] and https://www.drugs.com [26]. Brown circles indicate the nitrogen atoms responsible for lysosomotropism; the orange circles indicate the strongest basic nitrogen atoms in photosensitizing compounds or its metabolites. pKa of the strongest basic nitrogen [27]: duloxetine 9.7, sertraline 9.85, amitriptyline 9.76, ciprofloxacine 8.68, linagliptin 9.86, lisinopril 10.21, metoprolol 9.67, terbinafine 8.86, pregabalin 10.23, ranolanzine 7.17, loratadine 4,33, desloratadine 9,73, amiodarone 8.47, minocycline 8.25, and doxycycline 8.33. High therapeutic dosage (e.g., terbinafine 250 mg/d, ranolazine 750 mg twice per day) may compensate for poor lysosmotropism. (**A**) Drugs with confirmed (blue) and supposed lysosomotropism (*). (**B**) Metabolites of drug with supposed lysosomotropism. (**C**) Photosensitizing drugs hypericin and prominent tetracycline antibiotics with protonatable basic nitrogen atoms, but without lysosomotropism [28]

Lysosomotropic drugs (Fig. 1) are small molecules accumulating in the lysosome usually by passive diffusion across the lysosomal membrane. They are characterized by one or more easily protonatable aliphatic nitrogen atoms localized in side chains or saturated ring systems, possessing a ClogP > 2 (lipophilicity), and a basic pKa between 6.5 and 11 [4, 5]. In the mild acid environment of the lumen of lysosomes, they become protonated and trapped, thus accumulating in the lysosome. Acyl amides and aromatic amines hardly exhibit lysosomotropism [25]. In contrast to the N-desmethyl metabolite desipramine, the hydroxy metabolites of imipramine (2-hydroxyimipramine and 10-hydroxyimipramine) exhibit no lysosomotropism [6].

Lysosomotropism is a biochemical characteristic of small compounds, independent of the individual, and fails to explain the fact that lysosomotropic compounds trigger more or less severe cutaneous adverse reactions. Chloroquine, amiodarone, chlorpromazine, amitriptyline, and trimipramine are classified both as photosensitizing [3] and lysosomotropic [6], however, they fail to trigger rash and pruritic papules in each individual.

A clue to the possible trigger of AGEP has been given by observed cutaneous adverse effects during topical application of amitriptyline on skin suffering from atopic dermatitis (AD) [29]. Contrary to expectations [30], severe pruritus with subsequent emerging papules and blisters have been observed while vanishing on discontinuation. Likewise, lysosomomotropic sertraline and terbinafine trigger pruritic papules vanishing on discontinuation of oral application [31, 32]. These findings imply that pruritic papules are apparently associated with the lysosomotropism of drugs and suggesting that lysosomotropism is a contributing factor, but not the determinant.

## Ceramides

**Fig. 2:**
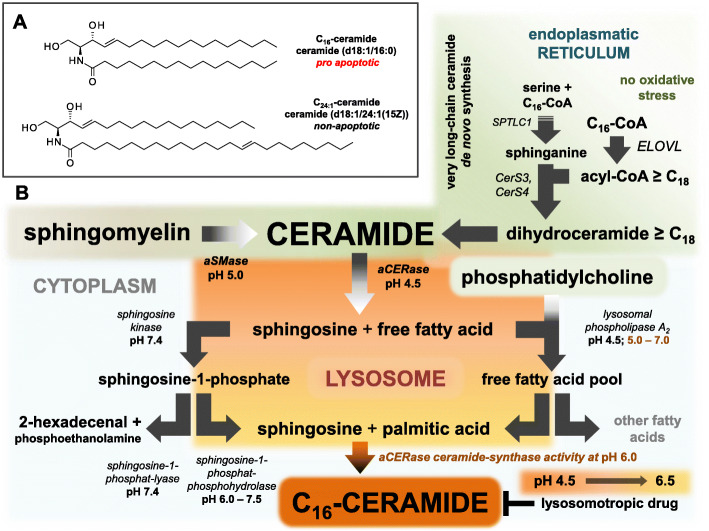
(**A**) Cell cycle-relevant, pro apoptotic long-chain C_16_-ceramide [34] and very long-chain C_24:1_-ceramide with sphingosine backbone and non-hydroxy fatty acid residue (ceramide fraction subclass NS representing 7% of cutaneous total ceramide [37]). A lack of very long-chain ceramides is responsible for loss of the barrier function of the stratum corneum [35, 38]. (**B**) Repercussions of lysosomotropic drugs and ceramide metabolism in keratinocytes. In standard conditions, ceramide de novo synthesis is localized in the endoplasmatic reticulum (ER) and ceramide degradation in lysosomes. Active pathways are marked with black arrows, and affected pathways are marked with arrows in shades of gray or red. Inhibition of acid sphingomyelinase (aSMase) and acid ceramidase (aCERase) is depending on the elevation of the lysosomal pH by the drug (strength of lysosomotropism and dosage). Enzymatic activity of aSMase and aCERase is strongly reduced, while residual activity remains for lysosomal phospholipase A_2_ [39]. Very long-chain ceramides (e.g., C_24:1_-ceramide) from the de novo synthesis at the ER accumulate in the cell membrane and the lysosomal synthesis of pro apoptotic long-chain C_16_-ceramide is blocked [25]. In presence of pronounced NADPH (and NADH/ATP depletion), lysosomotropic compounds are unable to prevent the formation of C_16_-ceramide and C_18_-Ceramide, if stearic acid is present (see Fig. 3)

Ceramides are well-characterized sphingolipid metabolites and second messengers in the cells, consisting of a backbone (dihydrosphingosine (sphinganine) [dS], sphingosine [S], phytosphingosine [P], or 6-hydroxy-sphingosine [H]) and a fatty acid residue (non-hydroxy fatty acid [N], 2-hydroxy fatty acid [A], or esterified ω-hydroxy fatty acid [EO]) [33]. Cell cycle-relevant, pro apoptotic C_16_-ceramide [34] and C_24:1_-ceramide belong to the subclass [NS], representing 7% of total cutaneous ceramide (Fig. 2A). Acyl residues of cutaneous ceramides typically range from C_16_ to C_26_, in the stratum corneum up to C_32_ [35]. On the basis of their fatty acid residue, ceramides can be classified in various categories: long-chain ceramides (C_14_-C_18_/C_20_), very long-chain ceramides (C_20_-C_26_), and ultra long-chain ceramides (> C_26_) [36]. Prominent C_16_-ceramide and C_18_-ceramide belong to the class of long-chain ceramides, C_24:1_ ceramide belongs to the very long-chain ceramides.

**Fig. 3:**
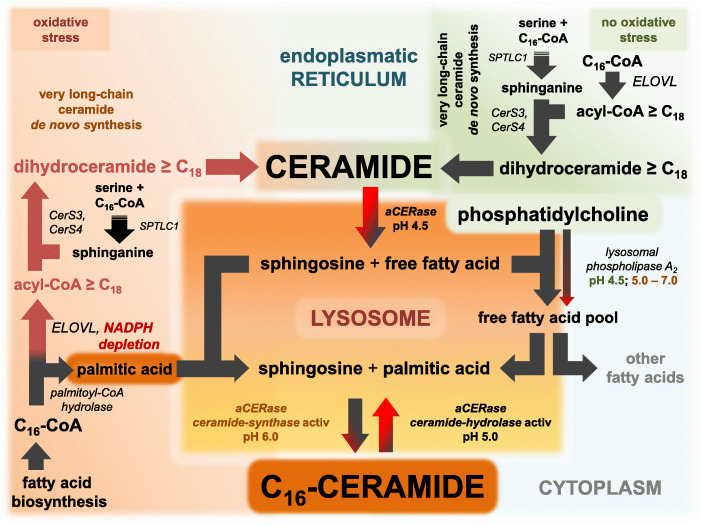
Ceramide metabolism in standard conditions and in oxidative stress. Active pathways at standard conditions are marked with black arrows, black and red shading arrows indicate pathways with diminished activity, and inactive pathways are marked with red arrows. In keratinocytes lacking NADPH, very long-chain fatty acid synthesis is impaired. C_16_-CoA is not converted to acyl-CoA ≥ C_18_ and accessible to other reactions, e.g., hydrolysis to palmitic acid. Natural and artificial UV radiation or ROS triggered severe depletion of NADH and/or ATP affects the maintaining of the lysosomal pH by the vacuolar (H^+^)-ATPase) and the lysosomal RedOx-chain. Raising the intralysosomal pH activates the reverse ceramide synthase activity of aCERase and the selective lysosomal synthesis and accumulation of C_16_-ceramide [40, 41] starting from palmitic acid and sphingosine. If stearic acid is present, C_18_-ceramide emerges likewise [40]. Alternatively, free palmitic acid in the ER can be converted to 2-hydroxy palmitic acid by fatty acid 2-hydroxylase (FA2H) [42] and recessed into ceramides in place of palmitic acid. Thereby, C_16_ (2-hydroxy) ceramide could result, which is significantly increased in lesions of AD [37]. Emerging C_18_-ceramide is assumed to trigger exocytosis [21]. C_16_-ceramide and C_18_-ceramide is increased in non-lesional and lesional atopic eczema stratum corneum [37] and hence supposed to trigger erythematous rash and pruritic papules. Instead of severe depletion of NADH and/or ATP, lysosomotropic drugs (e.g., amitriptyline and sertraline) or metabolites (e.g., nortriptyline) are likewise able to raise the lysosomal pH. With unimpeded ELOVL fatty acid elongation, there is limited palmitic acid present to synthesize C_16_-ceramide in the lysosome. On the other hand, in the event of an impaired ELOVL fatty acid elongation, excess C_16_-CoA is hydrolyzed to palmitic acid. Then, in the presence of lysosomotropic drugs in keratinocytes, C_16_-ceramide synthesis and accumulation can occur, probably leading to erythema, pruritus, and finally to acute generalized exanthematous pustulosis (AGEP). That implies that the elongation of very long-chain fatty acids (ELOVL, cofactor NADPH), maintaining of the lysosomal pH/proton gradient by the vacuolar (H^+^)-ATPase (V-ATPase, energy source ATP) and the lysosomal RedOx-chain (energy source NADH) are crucial parameters to retain the ceramide rheostat in keratinocytes. In addition to lysosomotropic drugs, a breakdown of the V-ATPase, e.g., by ATP depletion or formation of a disulfide bridge due to oxidative stress between the two cysteines at positions 254 and 532 of the P-LOOP of V-ATPase, leads to a collapse of the lysosomal transmembrane proton gradient. As a consequence of the collapse, C_16_-ceramide and the characteristic pruritic papules of PLE may occur. If excess C_18_-CoA is present, C_18_-ceramide can be formed via stearic acid under these conditions

## Ceramides and the barrier function of the stratum corneum

The stratum corneum is the outermost layer of the epidermis and responsible for barrier function against water loss. Extracellular domains are forming the barrier, consisting of protein-enriched cells (corneocytes with cornified envelope and cytoskeletal elements, as well as corneodesmosomes), and lipid-enriched intercellular domains [36, 43]. Distinct multi-lamellar membrane structures comprise a hydrophobic lipid mixture composed primarily of free fatty acids, cholesterol, and ceramides [36]. The balance of these components is important for maintenance of the protective role. Ceramide composition and lipid organization are associated with a reduced barrier function and transepidermal water loss (TEWL) in the stratum corneum of AD patients. In both, lesional and non-lesional skin of AD patients, TEWL is elevated [33]. Disease severity correlates with lipid composition, however, independent of filaggrin mutations [33, 37].

In AD, long-chain fatty acids (e.g., palmitic acid and stearic acid) and in particular the long-chain C_16_-ceramide are increased, while very long-chain fatty acids, and corresponding very long-chain ceramides (e.g., C_24:1_-ceramide) are decreased [33, 37]. In vitro studies on prototype lipid membranes of the stratum corneum composed of ceramides/free fatty acids/cholesterol/sodium cholesteryl sulfate have demonstrated that replacement of C_24_-ceramide by C_16_-ceramide has an impact on the microstructure and barrier function. Membranes containing more C_16_-ceramide become significantly more permeable to water, resulting in higher TEWL [44] and demonstrating the vital role of very long-chain ceramides for the barrier function of the stratum corneum. Consequently, the balance of both ceramide subtypes in the stratum coneum are important for the cutaneous barrier function and should be recovered in lesional and non-lesional skin of AD patients.

## Lysosomotropic drugs in skin susceptible to atopic dermatitis

In cells, lysosomotropic compounds (e.g., NB 06) are conducive to accumulate very long-chain ceramides and to suppress the oxidative stress induced synthesis of C_16_-ceramide. Shifting the intralysosomal pH to 5.5/6.0 results in an inhibition of aCERase, a blocked lysosomal degradation of very long-chain ceramides, and finally in an enrichment in cells [25]. In the presence of moderately reduced de novo synthesis performance of very long-chain ceramides in keratinocytes of the non-lesional skin of atopic patients, lysosomotropic drugs can mimic the typical very long-chain ceramide enrichment [25] during keratinocyte differentiation [36] to gain the barrier function of the stratum corneum. By cutaneous application of lysosomotropic drugs the deficiency of very long-chain ceramides, the increase of long-chain ceramide C_16_-ceramide in (non-) lesional skin of atopic patients and its sequelae [33, 37] appears avoidable.

Contrary to expectations, this treat-to-target strategy with lysosomotropic amitriptyline [6, 25] on lesional skin of AD patients was unsuccessful. Rather than an improvement of the lesions and the complexion, severe rash, pruritus, and papule formation appeared after a few days [29]. On healthy skin and keratinocytes with unimpaired very long-chain fatty acids elongation and ceramide de novo synthesis amitriptyline is well-tolerated. A significantly higher dosage of amitriptyline (2% than 0.03%) provoked no severe adverse reactions [30]. Obviously, well-known alterations present in the stratum corneum ceramide and fatty acids profile in AD [33, 37], higher TEWL and TEWL itch severity correlation in senile pruritus patients [45], and murine pre-symptomatic eczema [46] provide evidence of that changes in the lipid metabolism contribute to the emerging rash, pruritus, and papule formation. The clash of lysosomotropism of drugs and an altered sphingolipid-metabolism in keratinocytes resulted in severe papule formation. However, with the concomitant use of linoleic acid, the severe cutaneous adverse effects outlined above were absent and recovery of lesional skin was achieved [29].

## Amitriptyline, aCERase, and lysosomal ceramide metabolism

In standard conditions, ceramide metabolism is distributed across several cellular compartments (Fig. 2B and Fig. 3). De novo synthesis of very long-chain ceramides is located at the endoplasmic reticulum (ER) [47], whereas the breakdown to sphingosine and free fatty acids is mainly located in lysosomes. Synthesis of long-chain C_16_-ceramide in response to cellular stress is independent of CerS and ceramide de novo synthesis at the ER [48]. Lysosomotropic compounds such as NB 06, however, interfere with stress related C_16_-ceramide synthesis [25] suggesting the lysosome as its primary source. Independently, keratinocytes exhibit basal activity of virtually differentiation-independent ceramide synthases CerS5 and CerS6 [49], providing a basal level of C_16_-ceramide for incorporation into complex lipids (e.g., sphingomyelins, cerebrosides, and gangliosides) at the ER and Golgi membranes [47, 50].

If oxidative stress collapses, the proton gradient across the lysosomal membrane generated by the vacuolar ATPase (V-ATPase) and the lysosomal RedOx-chain, acid ceramidase (aCERase) shifts its enzyme activity to the reverse ceramide synthase activity of aCERase (revaCERase) [40]. Once the intralysosomal pH increases, non-selective ceramide degradation transition into selective synthesis of long-chain pro apoptotic C_16_-ceramide and C_18_-ceramide without ATP consumption.

Ceramide-1-phosphate (C1P) is a lipid second messenger, stimulates cell proliferation [51], and a lysosome-independent source of free ceramides in presence of lysosomotropic compounds. The post-Golgi localized membrane-bound lipid phosphate phosphatases LPPs converted C1P without being specific for particular fatty acids [52, 53]. Given the missing specificity of LLPs [52] and hardly any specificity of ceramide kinase for ceramide species [54], participation of C1P in altered in lipid profile of AD patients is unlikely.

Like all lysosomotropic compounds, amitriptyline raises the pH in lysosomes [6, 25]. Although the pH in the lysosome is increased as with oxidative stress, there is only a limited synthesis of C_16_-ceramide present [25], possibly due to a lack of free palmitic acid. However, the lysosomal phospholipase A2 (LPLA2) exhibits a residual activity at pH 5.0 of 6.5 for the hydrolysis of phosphatidylcholine (Fig. 2B and Fig. 3) still capable to release both fatty acids [39] and provide them for synthesis of C_16_-ceramide and C_18_-ceramide. Lysosomotropic conditions affect further lysosomal enzymes such as aSMase, glycosylceramidase [55], and acid lipase (LAL) [56]. Sphingomyelin or glycosylceramide turnover via ceramide and the hydrolysis of triglycerides and cholesteryl esters are substantially diminished, resulting in an enrichment within the cell. Consequently, free palmitic acid originating from these lipids is significantly limited and lacking for lysosomal C_16_-ceramide synthesis.

## Lack in cellular NADPH and impairment of ELOVL fatty acid elongation

Ceramide de novo synthesis at the ER of keratinocytes is a multistep process involving ceramide synthases CerS3 and CerS4 [49, 57], very long-chain-3-oxoacyl-CoA synthases (ELOVL) and fatty acid synthase (FAS) [58]. Biosynthesis of very long-chain fatty acid moieties (acyl-CoAs) involves a two-step process starting with long-chain fatty acid synthesis (to C_16_-CoA) by FAS, followed by extension of the carbon chain of C_16_-CoA by very long-chain-3-oxoacyl-CoA synthases (ELOVL1-7) at the ER. Within the skin ELOVL7 (preferably C_16_-C_22_ acyl-CoA), ELOVL3 (especially C_18_ acyl-CoA), and ELOVL6 (chain extension C_16_-ICoA to C_18_-CoA) are of particular interest. NADPH activates ELOVL6 and ELOVL7 enzymatic activity up to 10-fold, however, is not a cofactor of ELOVLs. In contrast, NADPH is a cofactor of 3-ketoacyl-CoA reductase, responsible for the downstream reduction step, that activates ELOVL6 threefold [59]. In the absence of NADPH due to oxidative stress, elongation of C_16_-CoA stops and C_16_-CoA undergoes hydrolysis to palmitic acid by palmitoyl-CoA hydrolase (acyl-CoA hydrolase). Sources of oxidative stress include UV radiation, inactivation of ROS, insufficient quenching of ROS, and photosensitizing xenobiotics in the epidermis [60, 61]. Impairment in mitochondrial fatty acid oxidation to generate energy may additionally aggravate the conditions [62]. ELOVL3, unlike the other ELOVLs, is NADPH independent and prevents accumulation of C_18_-CoA formed by ELOVL6.

In case of a missing ELOVL3 activity, accumulating C_18_-CoA can be degraded to stearic acid likewise. Then both C_16_-CoA and C_18_-CoA become available for lysosomal C_16_-/C_18_-ceramide synthesis by aCERase and result in the increase of the corresponding long-chain ceramides [33, 37]. In combination with ELOVL3, ELOVL7 provides the specific acyl-CoAs for the predominantly present CerS3 in differentiated keratinocytes [49, 57]. The impact of impaired ELOVL very long-chain fatty acid elongation can be demonstrated in non-lesional and lesional stratum corneum of AD patients. There, very long-chain fatty acids (≥ C_24_) are substantially reduced, while shorter long-chain fatty acids, in particular palmitic acid (C_16:0_) and stearic acid (C_18:0_), are increased [37]. In mast cells of the stratum corneum in murine presymptomatic eczema, the increase of C_16_-ceramide is present [46], suggesting that palmitic acid is increased and an impairment of ELOVL fatty acid elongation is already present at the early onset of AD.

## Impaired ELOVL fatty acid elongation on ceramides and other lipids

Impaired ELOVL fatty acid elongation effects more than the de novo synthesis of (very) long-chain ceramides with non-hydroxy fatty acid [N]. Fatty acids are participating in the biosynthesis of phosphatidyl glycerol, cardiolipin, cerebrosides, or diacylglycerol; fatty acids related metabolism; ω--O-fatty acid esterification; and the lipoxygenase (LOX) pathway. Within the epidermis, fatty acid moieties are present in triglycerides, phospholipids, and glycosylceramide [43].

Acetylated ω-hydroxy fatty acids and 2-hydroxy fatty acids are prominent moieties of cutaneous ceramides [33, 63]. In the epidermis, both can be derived from corresponding unmodified fatty acids. Selective ω-hydroxylation of aliphatic hydrocarbon chains is allocated to the microsomal monooxygenases cytochrome P450 family 4 (CYP4F) member CYP4F22 [64, 65]. Subsequent esterification of ω-hydroxy fatty acids with predominantly linoleic acid results in ceramides with [EO] moiety, essential for the integrity of the epidermal barrier [63]. To date, the epidermal synthesis of EO ceramides has not been fully elucidated. A sequence of initial glucosylation of ceramide [N] by glucosyltransferase, followed by ω-hydroxylation, terminal acylation of glucosylceramide, and deglucosylation of acyl glucosylceramide by acid glucosylceramidase resulting in ceramide [EO] is considered very likely [66].

At the ER the 43-kDa integral membrane enzyme fatty acid 2-hydroxylase (FA2H) transforms fatty acids to 2-hydroxy fatty acids [67, 68], the [A] moiety of ceramides. Synthesis of 2-hydroxy ceramides is CerS-mediated, thus identical to biosynthesis of non-hydroxy ceramides [N], except for the fatty acid 2-hydroxylation step [67]. As part of keratinocyte differentiation, FA2H expression and production of free 2-hydroxy fatty acids increase first, followed by 2-hydroxy ceramides/2-hydroxy glucosylceramides with very long-chain fatty acid moiety [42]. Together with epidermal protein-bound ω-hydroxy ceramides [69], they are required for the assembly of the epidermal lamellar membrane. Thus, it is extremely likely that ELOVL fatty acid elongation effects the composition of all types of ceramides in the epidermis, the barrier function of the stratum corneum, and the TEWL. Interestingly, the lipoxygenase (LOX) pathway is linked likewise to barrier permeability, formation, and function [70].

In fact, changes in free fatty acid chain length distribution of 2-hydroxy fatty acids (chain length ≥ 18 carbon atoms) and very long-chain ω-hydroxy fatty acids (chain length ≥ 24 carbon atoms) are present in lesional and non-lesional stratum corneum of AD patients in comparison to healthy skin [37, 69]. As with non-hydroxy fatty acid fatty acids [33, 37], the subsidence both modified very long-chain fatty acids is already evident in non-lesional skin and particularly pronounced in lesional skin [37, 69].

## 2-Hydroxy palmitic acid and C_16_ (2-hydroxy) ceramide in AD

Among ceramides significantly altered in AD, in particular the long-chain ceramides C_16_-ceramide and C_16_ (2-hydroxy) ceramide (also referred to as C34 CER [NS] and [AS]) strikingly increase. Their common feature is the unbranched C_16_ fatty acid moiety. Given that palmitic acid can be converted to 2 hydroxy palmitic acid by FA2H [42, 67] at the ER, it stands to reason that, as with C_16_-Cer, the increase in C_16_ (2-hydroxy) ceramide is most likely associated with stress-induced impairment of ELOVL fatty acid elongation. If instead of palmitic acid, 2-hydroxy palmitic acid is reacted with sphingosine by revaCERase, C_16_ (2-hydroxy) ceramide is obtained, being significantly increased in lesions in AD [37]. Currently, studies on the substrate specificity of revaCERase with unbranched 2 hydroxy fatty acids are still lacking. Assuming that 2-hydroxy palmitic acid is a substrate of revaCERase equivalent to palmitic acid, lysosomotropic compounds may also cause an increase in C_16_ (2-hydroxy) ceramide in presence of impaired ELOVL fatty acid elongation.

## Photosensitization, ROS, ER stress, and lysosomal proton gradient breakdown

The perylenequinone hypericin, isolated from St John’s wort, is a potent photosensitizer that efficiently localizes in the ER and triggers ER stress after light application [71]. When sunlight interacts with photosensitizers such as hypericin in the stratum corneum, electrons in the photosensitizer get excited, creating unstable singlet or triplet states, which in turn oxidize subcellular structures and molecules [72]. This ER related stress is often accompanied by depletion of cellular NAD(P)H and ATP. Given that the lysosomal proton pumps utilize ATP (V-ATPase) and NADH (lysosomal RedOx-chain) from the cytoplasm as energy source [73, 74], ER stress is affecting both lysosomal proton pumps, leading to an increased lysosomal pH.

Furthermore, hypericin-induced ER stress may also decrease the NADPH-dependent ELOVL fatty acid elongation at the ER. By impairment of ELOVL fatty acid elongation, more C_16_-CoA is available for hydrolysis to palmitic acid and finally for C_16_-ceramide synthesis in lysosomes (Fig. 3). The characteristic pruritic papules of PLE filled with sterile liquid (plasma) may develop. The tetracyclic antibiotics doxycycline, minocycline, and tetracycline (Fig. 1C) are likewise potent photosensitizers [71, 75] able to interfere with sphingolipid metabolism via singlet oxygen mediated oxidative stress. Doxycycline, for example, provokes mitochondrial stress and ATP depletion, which can be counterbalanced more or less depending on the cell line [76], suggesting that the capability of stabilizing the mitochondrial fatty acid oxidation and thus the energy balance varies between cell lines.

## Papules of polymorphous light eruption (PLE) linked to V-ATPase, ELOVLs or both?

Hypericin lacks lysosomotropism, nevertheless it is capable of triggering the formation of papules, in particular in presence of intense UV radiation similar to lysosomotropic drugs. Additional to lysosomotropism of drugs, the collapse of the lysosomal transmembrane proton gradient and the rise of lysosomal pH can be triggered by further factors. First, oxidative stress-induced depletion of NADH and ATP has an impact on the energy supply of both proton pumps and exacerbates to maintain the transmembrane proton gradient. Second, the catalytically active subunit (73 kDa) of V-ATPase contains a conserved region (P-LOOP) with two cysteines at positions 254 and 532, capable of forming disulfide bonds and thus inactivating the active site of V-ATPase [73]. Since the thiol-disulfide equilibrium is linked to the redox potential of the cytoplasm, a severe depletion of the cytosolic redox potential results in formation of the disulfide bond between Cys 254 and 532, blocking the catalytically active site of the V-ATPase. Once formed, the disulfide bond is quite durable, since physiological cytoplasmic GSH concentrations fail to reconstitute the cysteines and recover full V-ATPase activity [77]. It is suggests that formation of the disulfide bond is probably not the cause of V-ATPase failure but more probably due to presence of severe ROS stress.

Prior to disulfide linkage in V-ATPase, xenobiotic- or UV-radiation-induced stress affects both NADPH-dependent ELOVL6 and ELOVL7. Termination of ELOVL fatty acid carbon chain extension and shift to long-chain fatty acids affects lipid composition, however, inconclusively results in pathological changes in skin. Examples include ceramide profiles from non-lesional atopic eczema stratum corneum [37] and mast cells in presymptomatic eczema [46]. This suggests that, failure of V-ATPase outweighs impairment of ELOVLs in development of pruritic papules. Only in coincident presence of both alterations, preconditions for development of pruritic papules of PLE apparently exist.

## Dual-functionality within one compound

In contrast to hypericin, tetracyclines are supposed to have a dual-functionality due to their chemical structure and characteristics. They are, in addition to being effective generators of singlet oxygen mediated oxidation [72], likewise probably lysosomotropic by virtue of a protonatable nitrogen in aliphatic 4-dimethylamino residue (pKa 8.33, doxycycline). Since calculated lipophilicity of doxycycline (ClogP − 0.5) is inadequate, doxycycline lacks like many other polyhydroxylated fused rings and tetracyclines lysosomotropism [28, 78]. In presence of UV light, the photosensitizing characteristics of tetracyclines trigger similar to hypericin immediately or time-delayed (1–2 days) ROS-induced photodermatitis (erythema, pruritus, and vesiculation) [3]. Tolerance to phototoxicity and resultant photodermatitis of doxycycline decreases in a dose-dependent manner from 97% (100 mg/d) to 58% (200 mg/d) [79]. In severe cases (e.g., UV light induced strong NAD(P)H/ATP depletion), even necrosis is possible [80].

## Accidental concurrent application of lysosomotropic and photosensitizing drugs

Distinct photosensitizing characteristics and lysosomotropism together in one active compound is exceptional, however, the encounter of two or more (active) compounds displaying either of them in the skin is very likely. Duloxetine is a lysosomotropic selective serotonin noradrenaline reuptake inhibitor (SSNRI) [7], frequently used for treatment of major depressive disorder, diabetic peripheral neuropathic pain, and generalized anxiety disorder. Systematic administration is often accompanied by cutaneous adverse reactions (Table 1) such as rash; urticaria, contact dermatitis, photo-sensitivity reactions; and Stevens-Johnson Syndrome (post-marketing surveillance reported adverse reaction) [81].

In AD-prone individuals, ELOVL fatty acid elongation is mostly impaired already, however, the proton gradient across the lysosomal membrane is still present. Adding duloxetine collapses the lysosomal proton gradient in keratinocytes and C_16_-ceramide can be formed in lysosomes, which in turn can induce cyclooxygenase 2 (COX2) expression [82] and affect arachidonic acid metabolism. Thus, AD patients may experience well-known cutaneous adverse reactions of duloxetine more frequently and more seriously. However, simultaneous oral or topical application of drugs or food containing potent photosensitizer such as hypericin in addition to duloxetine is expected to increase the severity of cutaneous adverse reactions likewise, in particular in presence of intense UV radiation.

During concomitant use of duloxetine and herbal preparations containing St John’s wort (Hypericum perforatum) cutaneous adverse reactions may be more common. Hence, a warning has been included in the product information of duloxetine containing pharmaceuticals [8].

## Unsaturated fatty acids are promising options to alleviate and protect from PLE and AGEP

In sum, there is considerable evidence suggesting the involvement of the lysosome and ELOVL fatty acid elongation in the pathogenesis of AGEP and PLE. Both disorders apparently are the result of an impaired ELOVL fatty acid elongation plus an increased pH in the lysosome, either caused by lysosomotropic drugs or by a gradual failure of one or both proton pumps of the lysosome. The objective of preventive or therapeutic measures in PLE is to restore ELOVL fatty acid elongation and the lysosomal transmembrane proton gradient. Adequate supply of NADH and ATP allows the lysosomal proton pumps to re-establish lysosomal pH of 4.5-5.0 and aCERase activity. Furthermore, sufficient NADPH is present for ELOVL fatty acid elongation [83, 84]. In contrast to PLE, restoring ELOVL fatty acid elongation is the exclusive objective in AGEP, given that the lysosomotropic drug is necessary for therapeutic reasons and cannot be discontinued.

The major source of NADPH is the oxidative branch of the pentose phosphate pathway [85]. However, mitochondrial fatty acid oxidation is equally involved. Inhibition of fatty acid oxidation impairs NAD(P)H production and increases free ROS resulting in ATP depletion [62]. To compensate for the shortfalls, an increased mitochondrial fatty acid oxidation may provide more NADPH for backstop against oxidative stress and prevention of ATP loss. Naturally occurring polyunsaturated fatty acids (PUFA) are endogenous PPAR ligands [86]. In particular, linoleic acid is rated as an efficient ROS quencher, a PPARG activator [87], and finally an activator of fatty acid oxidation and so the means of choice.

## Suggested management of cutaneous adverse reactions AGEP and PLE

In the event of cutaneous adverse reactions of therapeutically required drugs (Table 1), the question arises whether the therapeutic benefit outweighs the severity of adverse reactions or an alternative, well-tolerated drug is available. Once e.g., amine oxidase (AO) or UV radiation induced ROS [88, 89] are inadequately quenched and stress at the ER causes NAD(P)H depletion, the likelihood of impairment of ELOVL elongation and the lysosomal proton pumps, and consequently the risk of developing scattered pruritic papules or AGEP increases. Simultaneous cutaneous application of PPARG activators such as linoleic acid provides the possibility to normalize NAD(P)H and ATP levels in keratinocytes in a convenient way and to enable the use of lysosomotropic drugs in individuals prone to cutaneous adverse reactions of drugs accumulating in the epidermis.

Local application of a lysosomotropic drug (e.g., amitriptyline) to the skin of individuals with (UV-) light-sensitive skin (prone to rash, itching, and AD) can provoke severe cutaneous adverse reactions (sever rash, pruritus, and papule formation). Co-administration of linoleic acid can be used successfully to prevent and repress these cutaneous adverse reactions [29]. Obviously, with the help of linoleic acid ATP and NAD(P)H levels in keratinocytes can, thus, be returned to normal. ATP and NAD(P)H depletion in keratinocytes by unbalanced ROS appears to be an underlying cause of AGEP and perhaps of other, more severe adverse reactions of lysosomotropic drugs such as TEN where apoptotic and necrotic cells are present [13].

Given that in PLE, unlike AGEP, the increased pH in the lysosome is triggered by a lack of NADH and ATP, both the functionality of the lysosomal proton pumps and the ELOVL fatty acid elongation need to be restored. Again, relief can be expected by cutaneous application of linoleic acid acting as PPARG and fatty acid oxidation activator. NADH and ATP return to normal can serve as energy carriers for the two lysosomal proton pumps, NADPH for ELOVL fatty acid elongation. In mild hypericin-induced photodermatitis, photosensitization, and PLE, this approach may offer a suitable preventive tool.

## Clinical relevance

Photodermatitis, erythema, (pruritic) papules, and AGEP are associated with a variety of common drugs (Table 1) and are accordingly omnipresent in medical practice and in pharmacies all over the world. So far, in most cases they can only be inadequately managed. Currently, suggested and frequently used oral or topical H_1_-antihistamines (dimetindene, diphenhydramine, ebastine and oratadine/desloratadine) in AGEP or pruritic papules [14] can be attributed to lysosomotropism or in case of diphenhydramine and loratadine rated as photosensitizers [3]. Sometimes they are more likely the underlying cause rather than a tool of an appropriate disease management.

PLE is common in Europe, in particular in Northern and Central Europe, affecting all skin types, mostly Caucasian and blonde [90]. Classified as a papular variant of PLE, Acne aestivalis (Mallorca acne) is an equally common monomorphic eruption consisting of multiple papular lesions and developing after sun exposure [91]. Both diseases lack effective prevention and therapeutic approach, besides conditionally effective topical corticosteroids and antihistamines [90].

Preparations of St. John's wort are commonly used as herbal antidepressants in the field of self-medication and prescription drugs. Photosensitizing characteristics of hypericin and concomitant impairment of ELOVL fatty acid elongation in the stratum corneum in the presence of UV light may increase the incidence and severity of cutaneous adverse reactions of lysosomotropic drugs (Fig. 1, Table 1). Skin prone to AD is similarly expected to have impaired ELOVL fatty acid elongation, resulting in an increased incidence of severe adverse reaction, similar to topical application of amitriptyline in lesional skin [29].

There is much evidence that unbalanced ROS, ATP and NAD(P)H depletion, and impairment of ELOVL fatty acid elongation in keratinocytes are common features of the specified cutaneous adverse reactions. Rebalancing ROS, ATP and NAD(P)H is a new target offering a new therapeutic option beyond topical steroids, antihistamines, or biologics (antibodies). In particular, topical linoleic acid preparations appear well suited in improving the tolerance of lysosomotropic drugs, UV radiation and sunlight; pruritic papules-inducing and photosensitizing drugs or preparations.

## Perspective

Previous mechanistic reviews on AGEP and PLE have been focused on the involvement of various immunocompetent cells, cytokines, and interleukins [1, 2, 13, 90]. However, changes in cutaneous sphingolipid metabolism and lipid profile, mitochondrial fatty acid oxidation, ELOVL elongation of fatty acids, and lysosomotropism of drugs have never been considered to be involved, although lysosomotropism is assumed or has been demonstrated for many approved drugs [5, 6, 92–94]. Both oxidative stress and lysosomotropism of drugs can cause a collapse of the proton gradient across the lysosomal membrane and an increase in pH inside the lysosome. The ability to respond to external oxidative stress by activating glycolysis, the pentose phosphate pathway, or mitochondrial fatty acid oxidation varies among individuals. Insufficient ROS compensation and NAD(P)H depletion has an impact on ELOVLs and the cutaneous composition of free fatty acids, ceramides, glycosylceramide,phospholipids, and triglycerides, which can be determined in the lipid profile.

Our model describing a compartmentalized ceramide metabolism provides a more sophisticated explanation of cutaneous drug adverse reactions and the individual sensitivity to UV radiation. Moreover, the model suggests measurable individual parameters such as cutaneous fatty acid profile and ceramide profile, and enables new concepts of scoring; in risk assessment of AGEP, PLE, and acne aestivalis; ﻿and evaluation of prophylaxis outcome. ELOVL impairment is supposed to correlate with the increase of long-chain fatty acids, appearance of C_16_-ceramide originating from the lysosome, and depletion of very long-chain fatty acids, suggesting ceramides and free fatty acids as useful biomarkers. With the likelihood of ELOVL impairment, an individual risk factor is proposed that allows better prediction of the individual risk of developing rash, erythema, and AGEP. Provocation testing and evaluation of ceramide and fatty acid profile might be useful in patients with a predisposition to PLE and acne aestivalis. In addition, cutaneous tolerance to photosensitizing or lysosomotropic drugs can be assessed in skin diseases associated with shifts in cutaneous ceramides and fatty acids, such as AD.

In summary, we believe that linking mitochondrial fatty acid oxidation and providing of NAD(P)H and ATP to lysosomal proton pumps, and in particular unimpaired ELOVL fatty acid elongation, are necessary for stable barrier function of the stratum corneum. To compensate existing shortfalls, PPARG activators such as linoleic acid are considered to be the means of choice.

## Data Availability

Not applicable

## Abbreviations

aCERase: Acid ceramidase
AD: Atopic dermatitis
AGEP: Acute generalized exanthematous pustulosis
C1P: Ceramide-1-phosphate
DRESS: Drug reaction with eosinophilia and systemic symptoms
ELOVL: Very long-chain-3-oxoacyl-CoA synthase
LAL: Lysosomal acid lipase
LPLA2: Lysosomal phospholipase A2
revaCERase: Reverse ceramide synthase activity of aCERase
PMLE/PLE: Polymorphous light eruption
ROS: Reactive oxygen species
TEN: Toxic epidermal necrolysis
TEWL: Transepidermal water loss
V-ATPase: Vacuolar (H+)-ATPase
